# High-Stability Hybrid Organic-Inorganic Perovskite (CH_3_NH_3_PbBr_3_) in SiO_2_ Mesopores: Nonlinear Optics and Applications for Q-Switching Laser Operation

**DOI:** 10.3390/nano11071648

**Published:** 2021-06-23

**Authors:** Siyu Dong, Cheng Zhang, Yuxiang Zhou, Xiaona Miao, Tiantian Zong, Manna Gu, Zijun Zhan, Duo Chen, Hong Ma, Weiling Gui, Jie Liu, Chen Cheng, Chuanfu Cheng

**Affiliations:** 1 College of Physics and Electronics, Shandong Normal University, Jinan 250014, China; dongsiyu929@163.com (S.D.); 13256715081@163.com (C.Z.); yx_zhou07@163.com (Y.Z.); xiaonamiao2021@163.com (X.M.); zongtiantian2020@163.com (T.Z.); gumanna1996@outlook.com (M.G.); zhanzijun1990@live.cn (Z.Z.); mahong@sdnu.edu.cn (H.M.); guiweiling@sdnu.edu.cn (W.G.); chengchuanfu@sdnu.edu.cn (C.C.); 2 International School for Optoelectronic Engineering, Qilu University of Technology (Shandong Academy of Science), Jinan 250353, China; sdkdcd@163.com

**Keywords:** perovskite, mesostructure, optical nonlinear, Q-switched laser

## Abstract

Hybrid organic-inorganic perovskite shows a great potential in the field of photoelectrics. Embedding methyl ammonium lead bromide (MAPbBr_3_) in a mesoporous silica (mSiO_2_) layer is an effective method for maintaining optical performance of MAPbBr_3_ at room temperature. In this work, we synthesized MAPbBr_3_ quantum dots, embedding them in the mSiO_2_ layer. The nonlinear optical responses of this composite thin film have been investigated by using the Z-scan technique at a wavelength of 800 nm. The results show plural nonlinear responses in different intensities, corresponding to one- and two-photon processing. Our results support that composites possess saturation intensity of ~27.29 GW/cm^2^ and varying nonlinear coefficients. The composite thin films show high stability under ultrafast laser irradiating. By employing the composite as a saturable absorber, a passively Q-switching laser has been achieved on a Nd:YVO_4_ all-solid-state laser platform to generate a laser at ~1 μm.

## 1. Introduction

Hybrid organic-inorganic perovskites (abbreviated to HOIPs) exhibit outstanding electronic, optical, and crystallographic properties, enabling high mobility and long carrier diffusion lengths [1,2,3,4,5]. They are promising candidates for photovoltaic applications and have rapidly achieved outstanding performances. As a member of this material category, CH_3_NH_3_PbBr_3_ is in the spotlight of research [6,7]. More recently, CH_3_NH_3_PbBr_3_ materials have been shown to be suitable for other photonic and optoelectronic device applications, such as light-emitting diodes, photodetectors, and lasers, which leverage their high photoluminescence efficiency and good electrical transport properties [8,9,10]. Based on these developments, we investigated the nonlinear optical properties of CH_3_NH_3_PbBr_3_ as a potentially important saturable absorber for ultrafast laser generation.

In recent years, it has been demonstrated that lead halide perovskites have excellent optical nonlinear properties, including third-order nonlinear absorption, saturable absorption, etc., which makes them suitable for numerous applications and devices. The nonlinear optical properties of materials can affect the performance of their photonic applications [11]. The investigation of ultrafast nonlinear properties (nonlinear susceptibility, nonlinear refraction and absorption, carrier relaxation lifetime, etc.) is critical for the development of 2D-material-based photonic and optoelectronic devices [12,13,14,15,16,17]. Nonlinear saturable absorption is essential for generation of ultrafast laser pulses, which has been widely applied in bulk, fiber, and waveguide laser systems [18,19,20,21,22,23]. Recently, the prominent third-order optical nonlinearities and saturable absorption performance of CH_3_NH_3_PbBr_3_ have been reported [24,25,26,27,28]. However, CH_3_NH_3_PbBr_3_ shows an unstable optical performance in the atmospheric environment. When it is exposed to the air, it generally easily decomposes into other components, which severely restricts the practical applications. In recent years, mesoporous structures embedding perovskite nanocrystals have been proved to be an effective approach to maintain perovskites in the atmospheric environment, by which many devices can be fabricated, including solar cells and photodetectors, etc. [29,30,31].

In our work, we designed a nano-composite structure in which CH_3_NH_3_PbBr_3_ was embedded in SiO_2_ thin film mesopores. We synthesized CH_3_NH_3_PbBr_3_ perovskite inside mesoporous silica templates covering a whole face of the substrate [32]. Fluorescence spectroscopy, absorption spectroscopy, small-angle X-ray diffraction (SAXS) and transmission electron microscope (TEM) were performed to characterize the features. The nonlinear responses and saturable absorption were measured by Z-scan technique under the excitation of femtosecond (fs) pulses at 800 nm based on the nonlinear optical properties of the nano-composite structure, and the applications as a saturable absorber (SA) for Q-switched lasers at ~1 μm wavelength. We developed the nano-composite as an optical switcher with remarkable stability, using it as a SA at 1 µm region for laser operation. All results indicate that embedding a nano-composite of CH_3_NH_3_PbBr_3_ in SiO_2_ thin film mesopores is an effective strategy to improve the stability and maintain optical performance of CH_3_NH_3_PbBr_3_ simultaneously, which provides a new insight into its practical applications.

## 2. Materials and Methods

### 2.1. Sample Preparation

**Figure 1:**
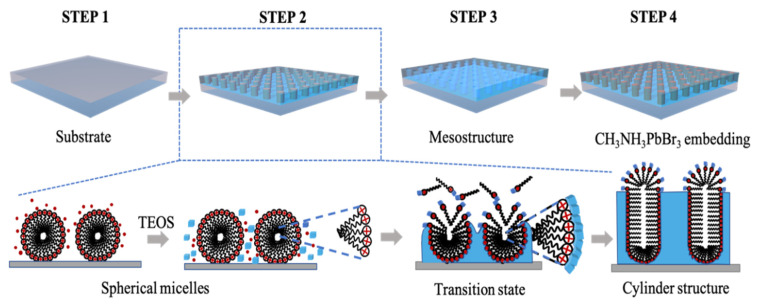
A schematic representation of preparation process of embedding the MAPbBr_3_ in a mSiO_2_ composite structure. Second line shows the formation process of ordered mesoporous silica films with perpendicular mesochannels by the Stöber solution spontaneous growth procedure.

The fabricating procedure of composites is shown in Figure 1. In the preparing procedure of mSiO_2_ and perovskites, ammonia aqueous solution (25 *wt*%), acetone, NH_4_NO_3_, tetraethyl orthosilicate (TEOS) and hexadecyltrimethylammonium bromide (CTAB) were purchased from Sinopharm Chemical Reagent Co. (Shanghai, China); lead bromide (PbBr_2_, ≥98.0%), methylammonium bromide (MABr, ≥98.0%), and *N*,*N*-dimethylformamide (DMF, ≥99.7%) were from Sigma Aldrich. Deionized water was used in all experiments. As the first line of this figure shows, steps 1 to 3 were the synthesis of mSiO_2_ thin films from pristine fused silica substrates. The mesoporous silica films were deposited on the substrates from a Stöber solution containing CTAB surfactant via a simple solution promoting spontaneous growth. A typical mixture consisted of 0.08 g of CTAB, 15 mL of ethanol, 35 mL of water, and 5 µL of concentrated ammonia aqueous solution (25 wt.%), to which 0.04 mL of TEOS was added under stirring. The substrate was then immersed in the precursor solution and film growth was achieved under quiescent conditions at 60 °C. After 48 h, the surfactant-templated silica films on the substrate were obtained and rinsed with water, and then dried and aged overnight in an oven at 100 °C. The surfactant template was removed by the solvent extraction method. The procedures of the template forming and removing are demonstrated in the second line of the figure.

The fourth step was embedding CH_3_NH_3_PbBr_3_ in mSiO_2_ thin films. The precursor was prepared by mixing an equal ratio of CH_3_NH_3_Pb and PbBr_2_ in DMF. The precursor was added to mesoporous silica films using the spin-coating method, then dried on a hotplate at 95 °C for 30 min before further characterization.

### 2.2. Characterization

For characterizing the structure and morphology of the mesopores and composite thin films, a transmission electron microscopy (TEM, NEC JEM-2100F, 200 Kv, Japan), a porosimetry system (Micromeritics ASAP2460, USA) and a small-angle X-ray diffraction (SAXS, Brucker D8 ADVANCE, Germany) system were utilized.

**Figure 2:**
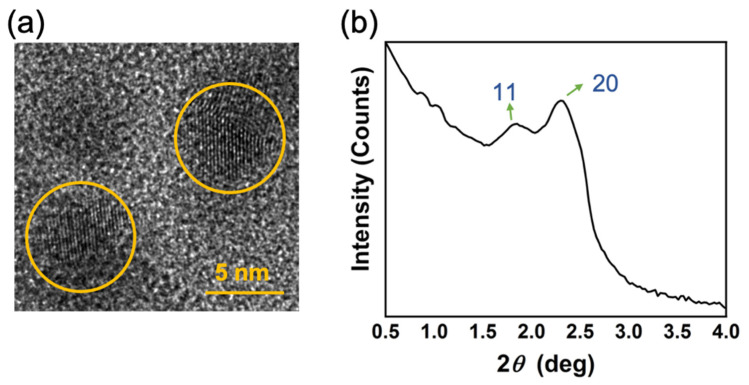
Structural analysis of the CH_3_NH_3_PbBr_3_ perovskite and mesoporous silica. (**a**) A TEM photograph of perovskite embedding in mesopores, with the perovskite quantum dots marked distinctly in the figure. (**b**) SAXS pattern of the perovskite embedding in mesopores.

According to the TEM images of CH_3_NH_3_PbBr_3_ embedding in mesoporous SiO_2_ thin films (Figure 2a), mesopores had been formed and were ready for embedding CH_3_NH_3_PbBr_3_ nanocrystals. TEM was used to image the CH_3_NH_3_PbBr_3_ nanocrystals encapsulated in the silica templates. The perovskite nanocrystal is notoriously sensitive to electron beam damage, so the TEM micrographs were taken with great care to minimize the exposure/dwelling times and avoid changes in morphology. After value calculation, the average mesopore size was ~4.4 nm. The TEM image shows that the nanocrystals were closely distributed along the channels embedding in the templates, which did not suffer from any deformation or collapsing.

The porosimetry system can be used to calculate the specific surface areas (SBET), using N2 adsorption data in a relative pressure range from 0.05 to 0.25, based on the Brunauer-Emmett-Teller (BET) method. The pore volume and pore size distributions were derived from the adsorption branches of isotherms using the Barrett-Joyner-Halenda (BJH) model. A few samples without embedding perovskites were tested using the system, in which the same synthesized conditions were set. After value calculation, the average mesopore size was ~3.5 nm, and the BET surface area and the total pore volume were 0.1205 m^2^/g and 0.014 mm^3^/g, respectively. Actually, the previous report noted that the mesopore size measured by SBET could be smaller than the size measured by other methods. The pore size distributions calculated from the N2 sorption isotherms with the BJH method can be matched with the particle size distribution measured by the TEM image.

The SAXS patterns (Figure 2b) of the templates containing CH_3_NH_3_PbBr_3_ can be observed. The peaks have been labeled in the figure. The previous work demonstrates that both peaks can be changed by the size of mesopores [30]. The pores exhibited periodic, long-range ordering, with peaks indexed to the (11) and (20) of a 2D hexagonal mesostructure (P6 mm). The result supports that the size of mesopores can be estimated to be ~4 nm, which can be matched to results of the TEM photograph and SBET.

**Figure 3:**
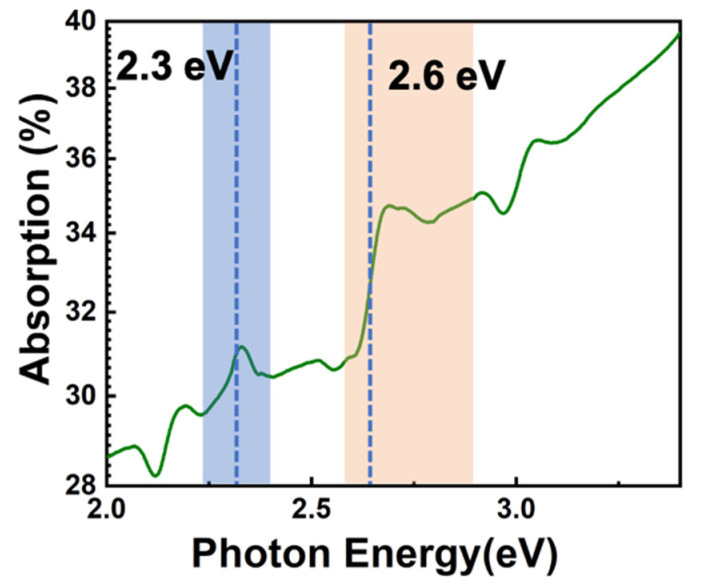
Absorption spectrum of perovskite embedding in mesopores as a function of conversion of photon energy. Working bands were labeled at 2.3 and 2.6 eV, respectively.

**Figure 4:**
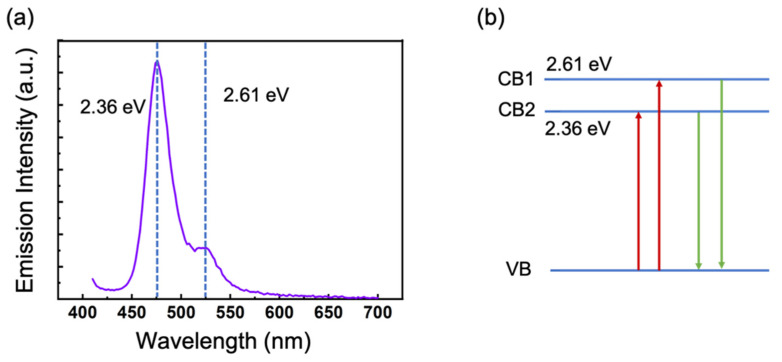
(**a**) PL spectrum of perovskite embedding in mesopores under 375 nm pumped emission. Two peaks appeared at 475 and 525 nm, which corresponded to 2.36 and 2.61 eV, respectively. (**b**) Schematic showing 475 and 525 nm photoluminescence and optical absorption.

To investigate the optical properties of samples, an absorption spectrometer was employed to explore absorption spectra of nano-composite samples. The absorption spectra of the samples were measured from 200 to 800 nm, with resolution of 0.2 nm. Figure 3 exhibits the linear absorbance as a function of conversion of photon energy. In this picture, two bands of 2.1~2.3 and 2.6~2.9 eV were labeled by colors. The energy level system corresponding to these two bands was mainly involved in this work. Furthermore, an ultra-fast spectrum system was used to analyze ultra-fast photo luminescence spectrum by a picosecond laser source with a wavelength of 375 nm, a 55 ps pulsed duration, and excitation power of 2.80 µJ/pulse. The corresponding luminance peaks of the samples were around 475 and 525 nm (Figure 4a). Because of the pore diameter of <5 nm, an obvious quantum confinement can be found. Figure 4b shows a schematic mark of a three-level system of PL processing, in which the optical absorption and photoluminescence processing of two excited states at 2.36 and 2.61 eV (corresponding to 525 and 475 nm emissions) are given.

## 3. Nonlinear Optical Properties

An open-aperture Z-scan system was employed to investigate the optical nonlinear performance of the nano-composite samples. An amplified femtosecond laser was used for testing optical nonlinearity with 35 fs pulsed duration, 1000 Hz repetition rate and 800 nm wavelength. A pair of lenses were used to focus the fs laser and collect response light with a focal length of 150 mm. The samples were mounted on a motorized stage to traverse the lens pair. The transmittance through the samples as a function of the incident intensity was detected by a photodetector located at the end of the system. The values of the incident laser energy were set to range from 40 to 90 nJ.

**Figure 5:**
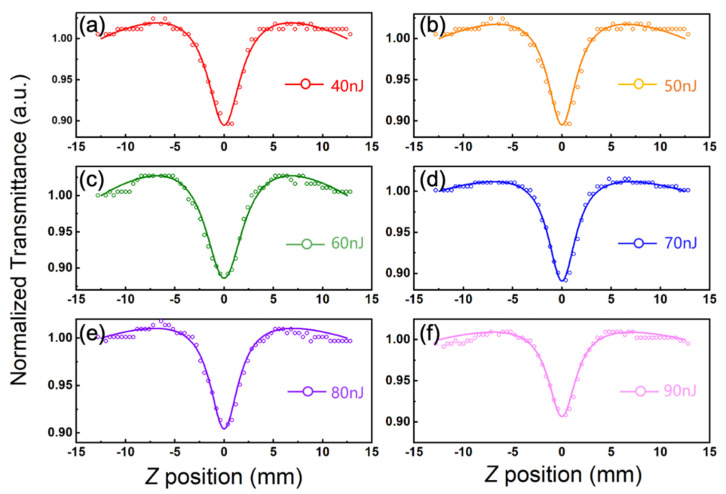
(**a**–**f**) The nonlinear optical properties of nano-composite samples measured by an open-aperture Z-scan system, with incident laser energy from 40 to 90 nJ.

Figure 5a–f demonstrates the typical Z-scan curves of the nanocomposite samples, exhibiting obvious nonlinear responses at 800 nm, under excitation pulsed energy from 40 to 90 nJ, respectively. The dots were the original detecting data. The data show a symmetrical undulating distribution to the center position (z = 0). With the positioning of z from the outside to the center position, the transmission ratio appeared to increase first and then decrease. The undulations indicate that two different absorption mechanisms may appear in nonlinear responses. Therefore, we used a formula that combines the existence of two mechanisms, including two-photon absorption and saturable absorption, to curve-fit the data. The formula is as follows [33,34],

(1) dIdz=−(α0+βI+11+IIsat)I

where *z* is the laser propagation distance in the sample, *α*_0_ is the linear absorption coefficient, *I*_s_ is the saturation intensity, and *β* is the nonlinear absorption coefficient. The solid lines were fitting curves.

**Figure 6:**
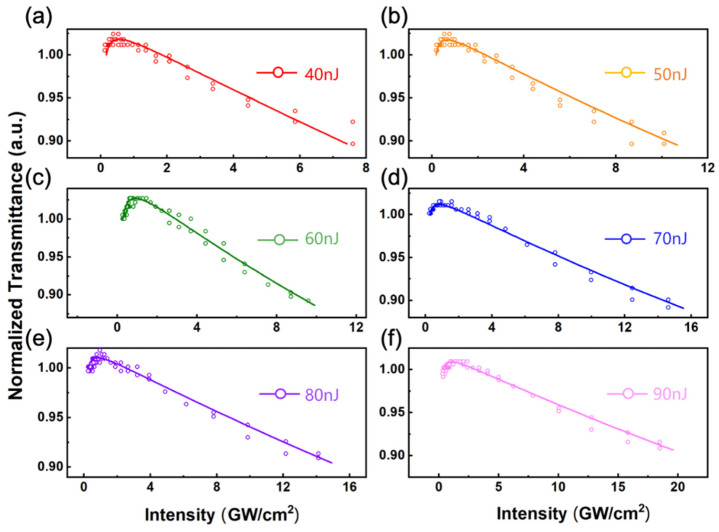
(**a**–**f**) Normalized transmission as a function of incident laser intensity from 40 to 90 nJ. The scatters are experimental data and solid lines are the fitting curves.

Figure 6a–f depicts the transmittance as a function of the excitation light intensity by considering the beam waist of the Gaussian beam. It is obvious that the transmittance of the low-intensity area caused by the saturated absorption of a single photon increases and the transmittance of the high-intensity area caused by the two-photon absorption decreases.

**Figure 7:**
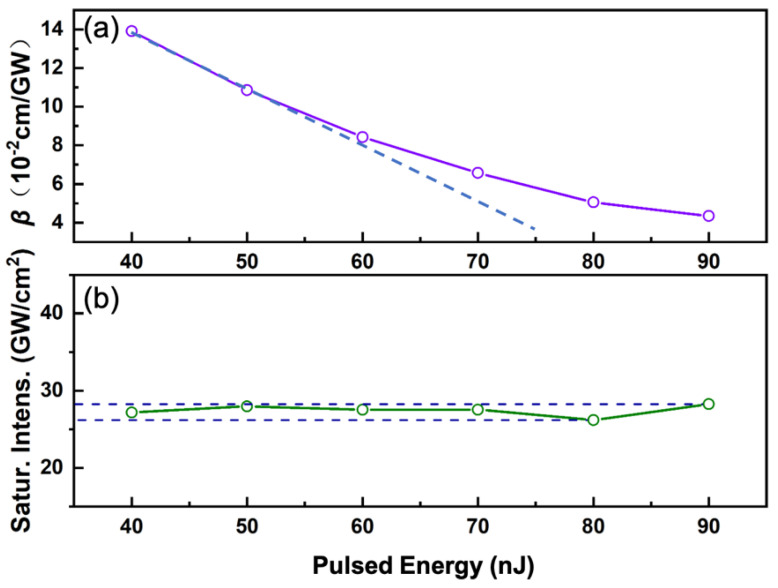
(**a**) Fitting values of nonlinear coefficient *β* and (**b**) saturation intensity as a function of the excitation pulsed energy.

After formula fitting, varying values of *β* were obtained and the positive values indicate a nonlinear response including two-photon absorption, as shown in Figure 7a. The value of *β* is a range from ~4.3 to ~13.9 × 10^−2^ cm/GW. In addition, the nonlinear absorbed coefficients exhibited the saturation effect. It is obvious that the rate of change of *β* tends to be flat with the increase of pulse energy. The blue dashed line depicts the initial slope of the *β* value, which as the pulse energy increases, then becomes significantly smaller.

Excluding the influence of substrate and the non-saturable loss, modulation depth and saturation intensity of the nano-composite samples were measured to be ~10% and 27.29 GW/cm^2^. It is worth mentioning that under normal circumstances, we think that the saturation intensity value reflects the intrinsic properties of the material. It is a specific value for the same material. After data fitting, we found that the value of saturation intensity was in a small range from 26.33 to 28.24 GW/cm^2^ (median was 27.29), shown in Figure 7b. Taking into account the complexity of materials and structural systems, we believe that fluctuations in the small value range of saturation intensity do not conflict with the previous understanding that the saturation intensity is a fixed value.

## 4. Q-Switched Laser Operation

**Figure 8:**
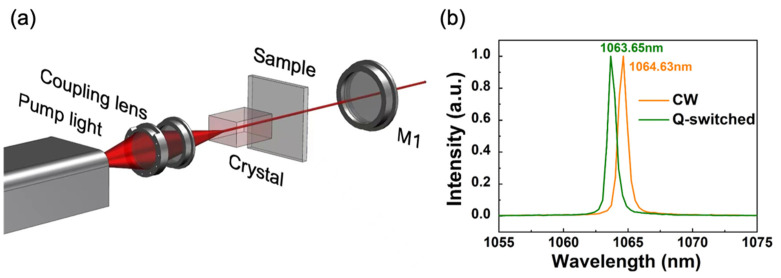
(**a**) The schematic of the experimental setup for the passively Q-switched operation. M1 was a concave dichroic mirror used as an output coupler. (**b**) Emission laser spectra of continuous wave (CW) and Q-switched laser modulated by perovskite nano-composition.

To test the potential applications of embedding perovskites in mSiO_2_ thin film, an all-solid-state Nd: YVO_4_ crystal laser system was utilized. In this system, the samples of perovskite embedded in mSiO2 thin film were regarded as a saturable absorber (SA). A schematic diagram for the CH_3_NH_3_PbBr_3_-mSiO_2_ SA pulsed laser is shown in Figure 8a. In this system, a 30 W commercial fiber-coupled laser diode emitting at 808 nm with a numerical aperture (NA) of 0.22 and a core diameter of 200 μm was used as the pumping source. The pump light was coupled into the Nd: YVO_4_ crystal by the optical collimation system of 1:1. The Nd:YVO_4_ (with 0.5 at.% Nd doping) crystal front end was plated with a high-reflective film for 1064 nm and a high-reflective film for 808 nm, and the rear end was plated with an anti-reflective film for 1064 and 808 nm. The dimensions of the crystal were 4 × 4 × 7.62 mm. The crystal was wrapped with indium foil and tightly mounted on a water-cooled copper heat sink at a temperature of 13 °C. M1 was a concave dichroic mirror having a radius of 100 mm with transmission of 5% for 1064 nm, used as an output coupler. The cavity length of the resonator was 40 mm. The nano-composite sample was placed in the cavity as the saturable absorber for the passively Q-switched laser. The laser pulse was recorded by a Tektronix DPO4104 digital oscilloscope (1GHz bandwidth) and a fast InGaAs photodetector with rise time of less than 175 ps.

In this system, a continuous wave laser operation and a Q-switched pulsed laser operation were achieved. Figure 8b shows both spectra of the output laser at ~1064 nm wavelength. It clearly denotes the laser oscillation line corresponds to the main fluorescence of ^4^F_3/2_ → ^4^I_11/2_ transition of Nd^3+^ ions.

**Figure 9:**
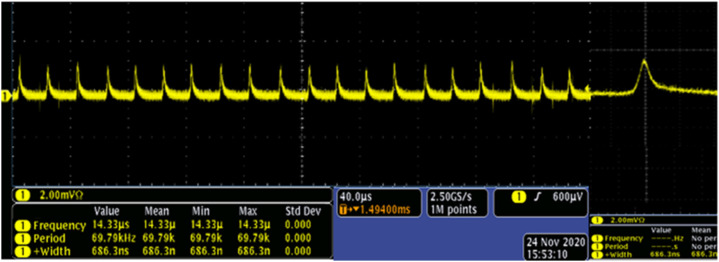
Oscilloscope display of passive Q-switched pulse trains at the maximum output power. The last one is a magnifying single pulse.

Figure 9 gives a description of the typical pulse trains at the maximum average output power captured by the digital oscilloscope (MDO4104C, Tektronix, Beaverton, OR, USA) with 1 GHz and a fast photodiode detector (ET-5000, Electro-Optics, Traverse City, MI, USA).

**Figure 10:**
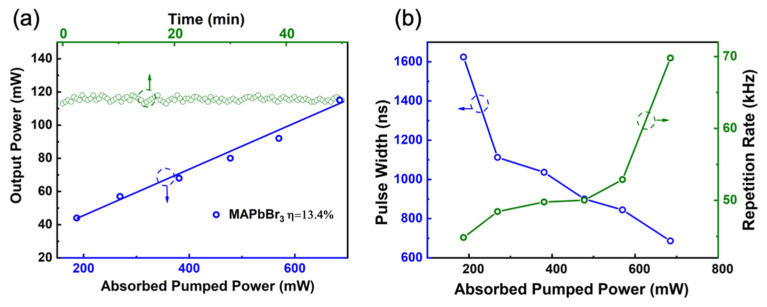
The performance of Q-switched laser operation including (**a**) average output power and instability variation with time; (**b**) pulse width and repetition rate as a function of absorbed pumped power.

In Figure 10, the repetition rate, pulse widths, average output power and instability of the laser are plotted for different transmissions. Figure 10a shows the experiment output power of the Q-switched laser operation, which is as a function of absorbed pumped power with the maximum value of 115 mW. The top axis corresponds to the stability of the Q-switched laser operation under maximum pumped power over 50 min. The value of power instability was estimated to be 4.2%. Figure 10b shows the experimental pulse parameters of the lasing, of which blue symbols and lines correspond to the left axis and green ones correspond to the right axis. When the absorbed pump power increased, the repetition rates gradually increased while the pulse widths decreased; the highest pulse repetition rate was 70 kHz and the lowest pulse width was 686 ns, corresponding to pumped power of 700 mW. The repetition rates of the Q-switched laser system were exhibited in a tunable range from 45 to 70 kHz as the absorbed pump power increased while the output power was steady with time, which shows the samples were stable in working condition.

## 5. Conclusions

In conclusion, we successfully fabricated high-stability CH_3_NH_3_PbBr_3_ embedding in mesoporous SiO_2_ thin film and investigated its optical nonlinearity by employing the Z-scan technique at 800 nm with femtosecond laser pulses. The thin film exhibited excellent optical properties with saturation intensity of ~27.29 GW/cm^2^ and nonlinear absorbed coefficients from 0.1391 to 0.0434 cm/GW with the increase in excitation peak power. In addition, the nonlinear absorbed coefficients exhibited the saturation effect. The thin film was employed as a SA to generate a Q-switched pulsed laser at 1.06 μm. The lasers worked stably with tunable repetition rates and nanosecond-level pulse durations. The results of the Z-scan and laser system indicated excellent nonlinear properties of the thin film. Our results suggest embedding CH_3_NH_3_PbBr_3_ in mesoporous SiO_2_ thin film can be applied as promising SAs in bulk or integrated active devices for ultrafast laser generation.

## Data Availability

Data are contained within the article.
